# Defining the role of digital public health in the evolving digital health landscape: policy and practice implications in Canada

**DOI:** 10.24095/hpcdp.44.2.04

**Published:** 2024-02

**Authors:** Ihoghosa Iyamu, Geoffrey McKee, Devon Haag, Mark Gilbert

**Affiliations:** 1 School of Population and Public Health, University of British Columbia, Vancouver, British Columbia, Canada; 2 British Columbia Centre for Disease Control, Vancouver, British Columbia, Canada

**Keywords:** digital public health, digital transformation, digitalization, public health, health equity

## Abstract

In this article, we argue that current digital health strategies across Canada do not appropriately consider the implications of digital technologies (DTs) for public health functions because they adopt a primarily clinical focus. We highlight differences between clinical medicine and public health, suggesting that conceptualizing digital public health (DPH) as a field distinct from, but related to, digital health is essential for the development of DTs in public health. Focussing on DPH may allow for DTs that deeply consider fundamental public health principles of health equity, social justice and action on the social and ecological determinants of health. Moreover, the digital transformation of health services catalyzed by the COVID-19 pandemic and changing public expectations about the speed and convenience of public health services necessitate a specific DPH focus. This imperative is reinforced by the need to address the growing role of DTs as determinants of health that influence health behaviours and outcomes. Making the distinction between DPH and digital health will require more specific DPH strategies that are aligned with emergent digital strategies across Canada, development of intersectoral transdisciplinary partnerships and updated competencies of the public health workforce to ensure that DTs in public health can improve health outcomes for all Canadians.

**Highlights**

- Current and emergent digital health strategies in Canada have not fully accounted for the application of digital technologies in achieving public health goals.
- A digital public health framing as a practice distinct from, but related to, digital health allows the public health workforce to develop digital technologies that will achieve public health goals while addressing contemporary challenges facing the field.
- The emergence of digital technologies as a determinant of health and health behaviours strengthens the argument for a digital public health framing and requires the public health workforce to develop new expertise to address both new and longstanding determinants of health.

## Introduction

The World Health Organization (WHO) defines digital health as “a broad umbrella term encompassing eHealth (which includes mHealth), as well as emerging areas, such as the use of advanced computing sciences in ‘big data,’ genomics and artificial intelligence”.^1^^,p.1^ In Canada, digital health is considered a broad field leveraging digital technologies (DTs) across clinical and community care, to optimize health outcomes and ensure connected, convenient, efficient and cost-effective person-centred care. With the expansion of digital health during the COVID-19 pandemic, many Canadian jurisdictions launched digital health strategies covering various health specialties, including public health.^2^ Most strategies emphasized the patient-provider-health systems interface, giving patients greater access to and control of their health data, while addressing longstanding issues such as access to primary care and wait times for specialist care.^2^,^3^ The Pan-Canadian Health Data Strategy was also launched to address requirements for common data policy frameworks and interoperability standards to allow data sharing.^3^ However, these strategies adopt overtly clinical perspectives, with none explicitly considering the role of DTs in public health.

Since 2017, when Public Health England launched its “digital first” strategy, the term “digital public health” (DPH) has been used to describe a distinct practice involving the application of DTs in public health functions.^4^,^5^ During the pandemic, this practice gained popularity, with utilization of DTs such as data analytics and dashboards for real-time disease surveillance, social media for health promotion and communication and apps such as Canada’s COVID Alert for infectious disease exposure notification and contact tracing.^6^,^7^

In our team’s scoping review of DPH, we found inconsistencies in its conceptualization and definition.^8^ Practitioners and researchers have either considered DPH as a tool to achieve existing public health goals, or as a response to wider societal digital transformation that demands a more fundamental integration of DTs with public health functions centred on the needs of communities and populations.^8^,^9^ However, DPH’s relevance to supporting public health efforts while upholding fundamental public health principles remains uncontested.^4^,^7^ While DPH is subsumed within digital health discourse in Canada despite its increasing prominence, we must consider the policy and practice benefits to be accrued from focussing on DPH as a distinct field alongside digital health. 

The current vague distinction between DPH and digital health reflects similar ambiguities between clinical medicine and public health.^10^ Clinical medicine emphasizes diagnosis and treatment of individuals, with responsibility to patients, albeit tempered by an awareness of their social contexts and health conditions.^11^ In contrast, public health focusses on the health of communities (at a population level), emphasizing health promotion, protection and prevention.^11^,^12^ Clinical medicine and public health are complementary fields with overlapping functions such as immunization, lifestyle modification (especially for chronic diseases) and disease screening.^10^ These overlaps may explain the current subsuming of DPH within digital health.^1^,^2^ Practitioners and researchers have also struggled to distinguish digital health from digital public health interventions.^13^

## The case for a distinction between digital health and DPH 

Differentiating DPH from digital health can help public health practitioners articulate and operationalize fundamental public health principles of health equity, social justice, ethics and action to address social and ecological determinants of health within their digital interventions.^7^,^14^ Many COVID-19 digital interventions were created using solely digital health perspectives in their design, implementation and evaluation, with public health principles being applied as an afterthought.^14^ While digital health interventions may be beneficial at the individual level, these benefits do not necessarily translate to equitable improvements in population-level health outcomes. Moreover, differences in digital access and literacy often determine the population subgroups that benefit from generically designed digital health technologies. We appreciate recent pivots in the digital health discourse to include health equity.^14^ However, the inherent responsibility to the patient underscored in the digital health (clinical) approach suggests health equity remains a secondary focus.

Further, “digital transformation” is a process accompanied by widespread societal adoption of DTs that influence health behaviours, access to health resources and health outcomes.^9^ These influences are recognized as “digital determinants of health,” and affect individual lifestyle, social, cultural and environmental determinants of health.^15^ This recognition expands previous, narrower views of digital determinants as being restricted to inequities in access to digital health interventions (i.e. differences in digital literacy and access) to include an understanding of how DTs are inequitably distributed in other facets of life, with direct and indirect effects on public health outcomes. The 2022 “NyQuil chicken challenge” (a popular trend in which social media users cooked and ingested chicken bathed in over-the-counter cold and flu medication) also demonstrates the public health risks DTs can pose and highlights the added health protection functions they require.^16^ Focussing on DPH can help public health researchers and practitioners develop the methods, skills and competencies required to understand the ramifications of digital determinants of health, while addressing them using fundamental public health principles. Such framings can also contribute to wider digital health interventions, especially interventions that consider health equity and social justice in their design, implementation and uptake, as universal interventions have been demonstrated to widen inequities.^7^

Moreover, widespread digital transformation has resulted in changing public expectations of public health services, which must ensure fast, responsive and convenient access to health information and services that are centred on their needs. These changing expectations are accompanied by new approaches to public health surveillance, with increased availability of new and diverse big datasets both within and outside of public health systems. Therefore, early and active participation of public health practitioners in the development of DPH can support resource allocation and development of nimble organizational processes to ensure that digital transformation of public health services appropriately optimizes public health outcomes.^9^ In our scoping review, we also found that conceptualizing DPH as a product of digital transformation requires practitioners and decision makers to embrace goals for interoperable, scalable and sustainable people-centred digital systems.^8^

Some may argue that distinguishing between digital health and DPH may be impractical and potentially perpetuates siloed programs and interoperability challenges limiting the potential impacts of DTs on health outcomes.^3^ DTs may also help transition health care from curative to preventive medicine, obscuring the demarcations between digital health and DPH.^4^ This transition towards preventive medicine through digital health might imply significantly greater contributions of public health practitioners to health interventions, with better resource allocation to achieve public health objectives. However, given inherent differences between DPH and digital health (Table 1), we anticipate that a broad view would result in inadequate attention to public health goals and functions. 

**Table 1:** Differences between digital health and digital public health

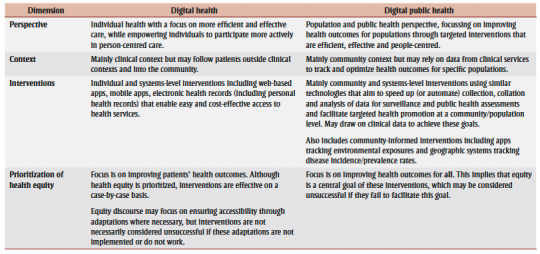

## Policy and practice implications 

Distinguishing between digital health and DPH has policy and practice implications, especially in Canada. Most emerging digital health strategies have a mainly clinical focus, only implicitly acknowledging the public health implications of DTs. Perhaps due to the provincial and territorial organization of health care, Canada has yet to develop a national digital health strategy—a key recommendation of the WHO global strategy for digital health.^1^ A DPH focus may inform strategies that advance the digital agenda in public health, applying systems thinking and approaches that ensure holism and perhaps a national strategy not only for harnessing health data but also for applying DTs to public health functions. Public health practitioners can also develop intersectoral and transdisciplinary partnerships (including partnerships with private sector organizations) to design and implement such strategies.^3^ Such strategies must consider public health perspectives in creating standardized ethical, regulatory and legal frameworks that are required not only to improve health equitably, but also to protect privacy and ensure the ethical use of available data.

While distinct, DPH strategies should be aligned and integrated with broader digital health strategies to fill gaps identified from a public health perspective. Such alignment might reduce the risk of siloed, vertical digital interventions that characteristically fail to meet public health goals. We must also evaluate the use of DTs within communities and populations, exploring and addressing their influence on public health behaviours as a health protection function. This perspective is largely missing from current discourse within digital health. Finally, the public health workforce must be better prepared to harness opportunities and address threats that DTs pose to public health. Competency frameworks must be updated to ensure the ever-expanding digital health and non-health data necessary for decision making for public health are better used to improve public health outcomes.^3^,^9^

## Conclusion

Distinctions between digital health and DPH are needed. Digital transformation spurred by the COVID-19 pandemic, changing public expectations about the delivery of health services, the increasing role of DTs in the determination of health and the threats they pose to population and public health in specific circumstances provide additional impetus for practitioners and decision makers to consider a specific focus on DPH. Explicit DPH strategies are needed to harness largely untapped potentials for DTs in public health. These strategies must be aligned with existing digital health strategies, drawing on cross-sectoral and interdisciplinary partnerships that emphasize evidence-based approaches to ensure health for all.

## Acknowledgements

II is supported by the Canadian Institutes of Health Research (CIHR) Frederick Banting and Charles Best Doctoral Award (Grant number AWD-018949 CIHR 2021), the University of British Columbia Four Year Doctoral Fellowship and the Bill Meekison Memorial Scholarship in Public Health.

***Funding***

This research received no specific grant from any funding agency in the public, commercial or not-for-profit sectors. 

## Conflicts of interest

The authors declare no competing interests.

## Authors’ contributions and statement

II, MG—conceptualization and literature review.

II—writing—original draft.

II, GM, DH, MG—formal analyses, writing—review & editing.

MG—supervision. 

The content and views expressed in this article are those of the authors and do not necessarily reflect those of the Government of Canada.
